# The Effect of Vitamin D Supplementation with or without Calcium on Vitamin D Epimer and Metabolites

**DOI:** 10.3390/metabo14100524

**Published:** 2024-09-27

**Authors:** Salah Gariballa, Ghada S. M. Al-Bluwi, Javed Yasin

**Affiliations:** Internal Medicine, College of Medicine & Health Sciences, United Arab Emirates University, Al Ain P.O. Box 15551, United Arab Emirates; ghadabluwi@uaeu.ac.ae (G.S.M.A.-B.); javed.yasin@uaeu.ac.ae (J.Y.)

**Keywords:** Vitamin D, calcium, epimers, metabolites, Vitamin D receptor, VDR

## Abstract

Background: A possible role of vitamin D epimers and metabolites in the measurement and response to treatment of vitamin D has been reported recently. Furthermore, the influence of underlying vitamin D receptor (VDR) genetic polymorphisms which have been linked to diseases such as obesity remains unclear. We therefore aimed to examine the influence of vitamin D3 and calcium supplements on vitamin D epimer and metabolite concentrations in subjects with and those without vitamin D receptor (VDR) gene polymorphisms. Methods: A total of 277 participants who were part of a randomized intervention trial of vitamin D3 and calcium or a placebo for 6 months had clinical and anthropometric assessments. Blood samples were taken for measurements of vitamin D, epimers and metabolites of vitamin D, four vitamin D receptor gene polymorphism SNPs, namely, BsmI, FokI, TaqI, and ApaI, metabolic and inflammatory markers, and related biochemical variables. Repeated-measures analysis of variance was used to assess the between-group difference in cumulative changes in vitamin D epimers and metabolites at 6 months after adjusting for the presence of the 4 VDR genotypes and allele gene polymorphisms. Results: Overall, 277 participants, with a mean (±SD) age of 41 ± 12 and 204 (74%) of whom were female, were included in the study. We found no statistically significant differences in vitamin D metabolites or (epimers) between male and females or younger subjects compared to those over 40 years of age except in 7C4 BL (*p* < 0.05). There was a statistically significant difference in 1,25(OH)2D3 concentrations between subjects with and those without genotypes AG and the allele G SNP2_Taql VDR gene polymorphism. Vitamin D3 concentrations were also significantly lower in subjects with the CC SNP3_Apal gene polymorphism compared to those without the CC SNP3 gene. No statistically significant effects were seen on vitamin D epimers and metabolites concentration in response to supplements before or after adjusting for the presence of the 4 VDR genotypes and allele gene polymorphisms. Conclusions: The CC SNP3 gene had statistically significant influence on vitamin D3 levels. Vitamin D and/or calcium supplements, however, had no effects on vitamin D epimer and metabolite concentration before or after adjusting for the presence of the 4 VDR genotypes and alleles.

## 1. Introduction

There is strong evidence that vitamin D has other health benefits because most human cells and tissues contain vitamin D receptors [1]. Vitamin D deficiency is common in the Middle East, but its adverse health effects and benefits of optimizing vitamin D status are still not clear. Vitamin deficiency may have a role in the pathologies associated with obesity and related diabetes [2]. Several observational studies have raised the possibility of the role of vitamin D in the development of type 2 diabetes in obese subjects [3,4]. This is important because the United Arab Emirates (UAE) has the second highest rate of obesity and associated type 2 diabetes in the world [5]. The biological action of vitamin D is through binding to vitamin D receptor (VDR) [1]. A number of mutations of the VDR gene have been identified [6]. In addition, several subtle allelic polymorphisms have been reported in the VDR gene, with links to metabolic bone diseases [7,8]. Polymorphisms and mutations of the VDR gene may influence the risk of disease and responses to circulating vitamin D, especially in the Asian and Middle Eastern populations where D deficiency is prevalent. However, studies on the relationship between vitamin D deficiency and its health implications on some ethnic groups have yielded conflicting results [9,10]. Some of these studies revealed only small ethnic differences in bone turnover, despite a striking difference in prevalence of secondary hyperparathyroidism between the two groups. The altered metabolism of vitamin D due to ethnic or genetic differences in Asian women may protect their skeleton from bone loss [11,12,13]. In addition, evidence points to a possible role of vitamin D metabolites in the measurement and response to supplements of 25-hyroxyvitamin D [14].

The main aim of this subgroup analysis was to examine the effects of vitamin D3 and calcium supplements on vitamin D epimer and metabolite concentrations.

## 2. Methods and Study Design

The methodology of this study has been published before [15]. Briefly, community free-living healthy subjects were recruited for this trial from January 2017 to December 2019. The subjects were recruited from community health centers and from hospital out-patient clinics. The exclusion criteria included those on vitamin D, calcium, and steroid medications, those with renal disease or stones, parathyroid disease, and hypercalcemia, and those unable to give a written informed consent. The trial was approved by the Al Ain Medical District human research ethics committee and all participants provided written informed consent. Clinical Trial Registration: NCT02662491, registered on 13 January 2016 (https://register.clinicaltrials.gov/prs/app/action/SelectProtocol?sid=S00060CE&selectaction=Edit&uid=U0001M6P&ts=3&cx=scu4cb (accessed on 11 September 2024)).

### 2.1. Study Design

Participants were assigned 2000 IU of vitamin D3, 600 mg of calcium, vitamin D3 (2000 IU) combined with 600 mg calcium, or a placebo daily for 6 months (enrollment flow diagram). Tablets in all 4 groups were identical in appearance and of equal size. Throughout the trial, all investigators were blinded to the intervention assignment. We assessed compliance by counting tablets at follow-up appointments. A computer random number table was used to generate the randomization sequence. Eligible subjects’ blood and urine samples were taken for measurements of 25(OH)D, markers of bone turnover, and related biochemical variables following written informed consent. An assessment of self-rated health, body pains, physical activity, dietary intakes, and bone turnover was performed at baseline, and repeated at 6 months post-randomization. Trial intervention tablets were stopped after the collection of the 6-month biological samples.

**Figure 1:**
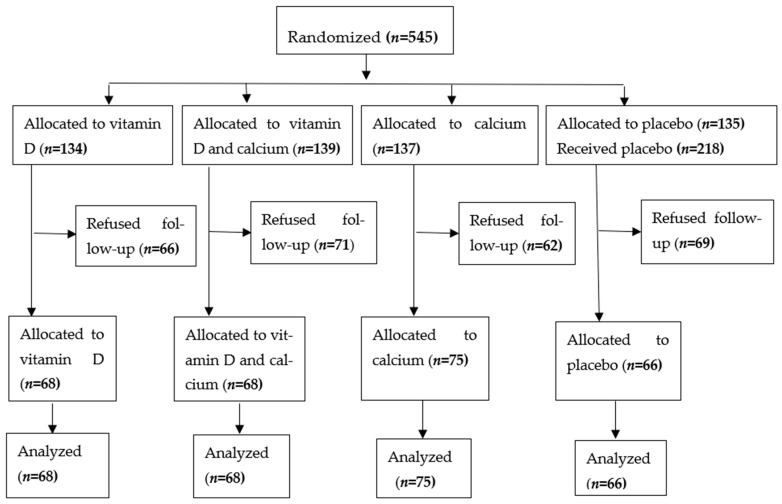
Enrolment, treatment and follow up of study subjects [15].

Enrolment flow diagram (Figure 1):

### 2.2. The Measurements

Details of the methods for clinical and biomedical measurements have been published previously [15,16,17]. Briefly, a questionnaire collected data on demographic and life style factors including the use of medications and supplements. Body mass index (BMI) was calculated from body weight and height measured using a Tanita body composition analyzer. Based on WHO cut-of-points for BMI, subjects with BMI = 18–25 were classified as normal weight, those with BMI = 25.1–29.9 as overweight, and those with BMI 30 and above as obese. 

#### 2.2.1. DNA Preparation and VDR SNP Genotyping Analysis [17]

This was described previously; briefly, DNA was extracted from blood using a QIAamp DNA Mini Kit. The extracted DNA was analyzed and stored at 80 °C until use. Four VDR SNPs (BsmI, FokI, TaqI, and ApaI) were evaluated using a TaqMan SNP genotyping assay. All assays were performed using Genetic Analyzer (Applied Biosystems, Foster City, CA, USA) according to the manufacturer’s instructions.

#### 2.2.2. Measurements of Vitamin D Epimers and Metabolites [17]

Vitamin D metabolites including 2 epimers were measured using the LC–MS/MS instrument (Shimadzu, Kyoto, Japan) [17]. The Shimadzu 8060 (Shimadzu, Kyoto, Japan) system was run using positive ion electrospray ionization (ESI). The method validation showed good sensitivity, recovery, linearity, precision, specificity, and accuracy [17].

### 2.3. Statistical Analysis

All data were analyzed using the SPSS V26. Testing for between-group differences was carried out using one-way ANOVA or the nonparametric Kruskal–Wallis H. Repeated-measures analysis of variance was used to assess the between-group difference in cumulative changes in vitamin D epimers and metabolites at 6 months after adjusting for the presence of the 4 VDR genotypes and allele gene polymorphisms.

## 3. Results

### 3.1. Baseline Characteristics

For those who completed 6 months of follow-up, baseline characteristics were well balanced across the groups except for the prevalence of diabetes (Table 1). Among the 277 subjects recruited, 46 (17%) had type 2 diabetes and 41 (15%) had hypertension. Using WHO obesity cut-of-points for BMI, 65 (24%) subjects had normal BMI, 93 (34) overweight, and 108 (39%) obesity at baseline (Table 1).

### 3.2. Vitamin D Epimer and Metabolite Concentrations Stratified by Age, Sex and Presence or Absence of the Genotype and Allele

**Figure 2:**
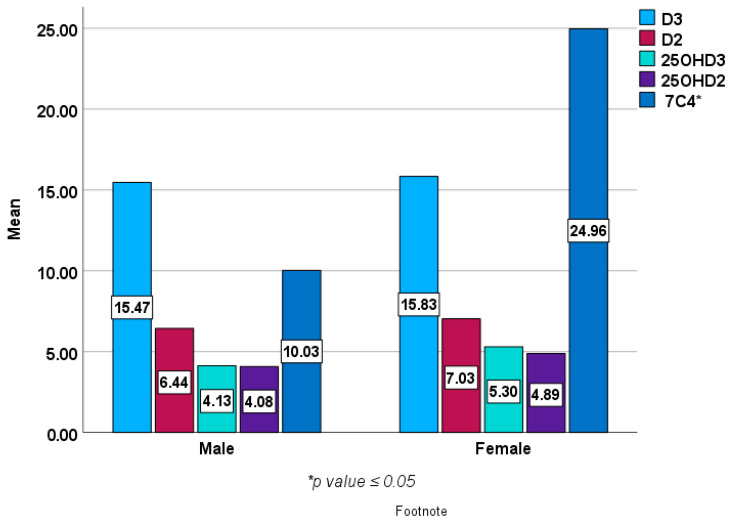
Baseline epimers and metabolites of vitamin D levels (ng/mL) in females (*n* = 203) compared with male subjects (*n* = 69).

**Figure 3:**
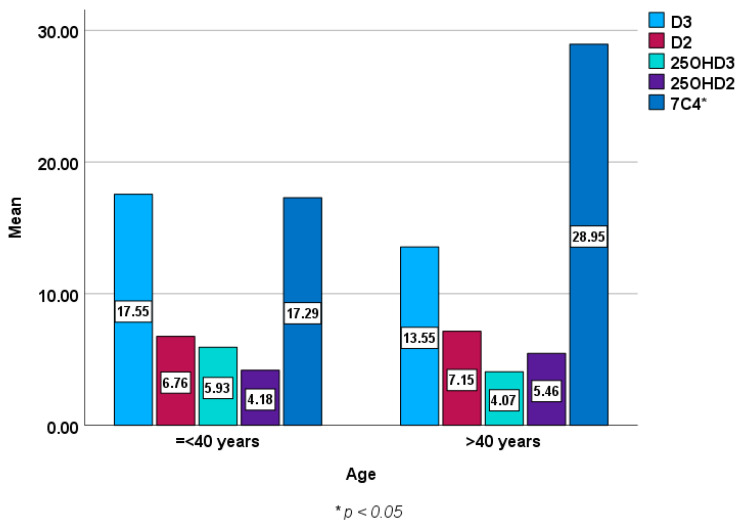
Baseline epimers and metabolites of vitamin D levels (ng/mL) in those aged ≤ 40 years [mean (±SD) age of 31 ± 7, *n* = 130] compared to those over 40 years [mean (±SD) age of 51 ± 8, *n* = 139].

Figure 2 and Figure 3 show baseline epimers of vitamin D in the study population stratified by sex and age over 40 years compared to those under 40 years of age. However, no statistically significant differences were seen in vitamin D or its metabolites (epimers) including 1,25 (OH)2D3 between males and females or between younger subjects and those over 40 years of age except in 7C4 BL (*p* < 0.05).

**Figure 4:**
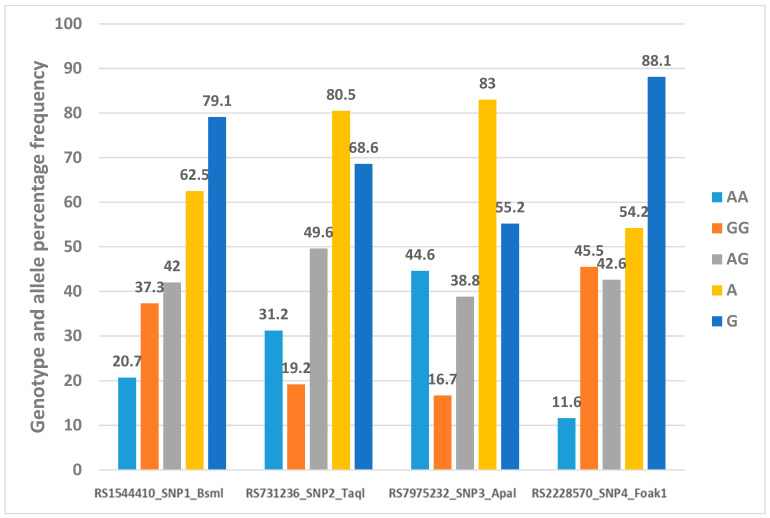
Genotype and allele percentage frequency distribution of 4 VDR gene polymorphisms in Emirati population [16].

Figure 4 shows the genotype and allele percentage frequency distribution of four VDR gene polymorphisms in 277 Emirati subjects.

Table 2, Table 3, Table 4 and Table 5 show vitamin D epimer and metabolite concentrations according to the presence or absence of the genotype and allele distribution of four VDR gene polymorphisms. There was a statistically significant difference in 1,25(OH)2D3 concentrations between subjects with and those without genotypes AG and allele G in the RS731236_SNP2_Taql VDR gene polymorphism (*p* < 0.05). Vitamin D3 concentrations were also significantly lower in subjects with the genotype CC in the SNP3_Apal gene polymorphism compared to those with and those without the genotype CC SNP3 (*p* < 0.05) [Table 4].

Table 6 shows the effect of supplements on vitamin D metabolites and epimers. There were no statistically significant differences in vitamin D metabolite and epimer levels between the supplement and placebo groups at the 6-month follow-up (Table 3).

## 4. Discussion

This is a subgroup analysis of the first randomized placebo-controlled trial to investigate the effect of vitamin D supplementation with calcium on vitamin D epimer and metabolite concentrations in subjects with and those without vitamin D receptor (VDR) gene polymorphisms. No statistically significant differences were seen in vitamin D metabolites or epimers between males and females or younger subjects compared to those over 40 years of age except in 7C4 BL. There was a statistically significant difference in 1,25(OH)2D3 concentrations between subjects with and those without genotype AG and allele G of the RS731236_SNP2_Taql VDR gene polymorphism. Vitamin D3 concentrations were also significantly lower in subjects with the genotype CC in the SNP3_Apal gene polymorphism compared to those with those without the genotype CC SNP3. No statistically significant effects were seen on vitamin D epimers and metabolites in response to supplements before or after adjusting for the presence of the four VDR genotype and allele gene polymorphisms. Several reasons may explain the lack of benefits of the supplements in our study [15]. First, there was a large number of recruited subjects who did not show up for follow-up. However, there were no statistically significant difference in baseline demographic and clinical characteristics between those who had follow-up data compared to those who did not come for the follow-up [15]. In addition, the pre-study sample size calculation shows that the number of participants with follow-up data is large enough to detect a difference if one exists. Second, vitamin D levels in the supplement group did not show the expected increase in relation to supplement dose and duration although this was slightly better in subjects who reported taking more than half of the prescribed trial medications. This could be a true finding or alternatively the result of the over-reporting of the number of tables taken although compliance was assessed by counting tablets at follow-up appointments in all study participants. Another reason which could explain the variability in response to supplements is the high proportion of overweight and obese subjects in our study population because overweight and obesity are known to markedly decrease response to vitamin D supplementation [18,19,20]. Recent guidance recommends obese subjects be given two to three times more vitamin D doses compared to normal-weight subjects [20]. Studies have reported different mechanisms for the poor response to supplements in obese subjects including decreased absorption, greater volume of distribution, and tightly bound vitamin D in fatty tissues; however, there is still no consensus on these mechanisms [20,21,22,23]. Our own society in the UAE has a growing epidemic of overweight/obesity, diabetes, and vitamin D deficiency. However, adjustment for factors known to influence vitamin D status such as body mass index, physical activity, sun exposure, and a vitamin D and calcium-rich diet did not show significant association with baseline vitamin D levels or response to supplements [17]. Recently, vitamin D supplementation to reach and sustain 25(OH)D levels ≥ 125 nmol/L has been shown to lower the risk of progression to diabetes in adults with prediabetes [24]. Accumulating evidence suggests that a higher dose of vitamin D supplementation to reach and sustain higher levels of 25(OH)D may indeed have clinical benefits in our high-risk population. 

A metanalysis of 76 trials of the influence of variable doses of vitamin D supplementation on serum 25-hydroxyvitamin D levels in Caucasians but not Asians reported that trials that used similar supplement doses could obtain significantly different changes to 25(OH)D concentrations [25]. In light of this evidence, the lack of significant increase in vitamin D levels or one of its metabolites or epimers in those who received the supplements in our study could be a true finding.

A number of factors have been reported to affect response to vitamin D levels and response to supplements including ethnicity, genetics, body mass, and type of vitamin D [1]. This may be the reason for the diversity in opinion reflected in different guidelines. Recent evidence also points to a possible role of vitamin D metabolites and epimers in the measurement and response to supplements of vitamin D [14]. The concentration of vitamin D epimers is reported to be greater in males than in females and the number of epimers was found to be higher in the blood of infants compared to adults [26,27,28]. It has also been reported that the oral supplementation of vitamin D can cause an increased production of epimers in mice but not in humans [29]. In addition, some experimental evidence suggests that adiposity reduces the hepatic hydroxylation of vitamin D, leading to lower vitamin D levels in obese mice compared with normal-weight mice [30]. Because of the controversy and difference in opinion regarding the clinical significance of vitamin D metabolites, we developed and validated the Ultra-High-Performance Liquid Chromatography-Tandem Mass Spectrometry (UHPLC-MS/MS) method and applied it to measure vitamin D metabolites in the serum of 452 obese and normal-weight healthy subjects [14]. Because of the increased risk of obesity-associated diabetes and vitamin D deficiency in our community and the paucity of evidence regarding the significance and storage effects of vitamin D status in obese subjects, we subsequently applied the above method to study vitamin D metabolites and epimers in a different cohort of community free-living healthy populations including obese subjects. We also studied the interaction and correlations of vitamin D and its metabolites with adverse metabolic health risk factors in obese subjects [2,17]. Although we reported a significant association between vitamin D levels and age, gender, and type 2 diabetes, we found no significant associations between the deficiency of vitamin D and or its metabolites and body mass index, inflammatory, or metabolic risk factors [17]. At present, clinical evidence does not support the routine quantification of vitamin D metabolites and epimers; however, our research has shown that vitamin D epimers are present in significant proportions in the blood, which can lead to an overestimation when measuring vitamin D levels [26]. The overestimation of vitamin D levels in patients undergoing vitamin D supplementation therapy is of serious concern, as sufficient vitamin D levels are critical for various important physiological functions and for disease prevention. 

### 4.1. Strengths and Limitations

Although this is a subgroup analysis of the effect of vitamin D supplementation with or without calcium on vitamin D epimer and metabolite concentration in subjects with and those without vitamin D receptor (VDR) gene polymorphisms, we acknowledge some limitations. 

One potential limitation is the lack of expected increase in vitamin D levels in relation to supplement dose used and duration, although this may be due to the high number of overweight and obese subjects in the study population or compliance with the trial medications. Compliance was assessed meticulously by counting tablets at follow-up appointments. 

### 4.2. Clinical Implications and Recommendations

Our previous research has shown that vitamin D epimers and metabolites are present in significant proportions in the blood, which can lead to an overestimation when measuring vitamin D levels [14]. Although clinical evidence at present does not exist for the routine quantification of vitamin D epimers and metabolites, the overestimation of vitamin D levels in patients undergoing vitamin D supplementation therapy is of concern, as sufficient vitamin D levels are critical for optimal health. It is therefore imperative for the healthcare community to be aware that reported vitamin D values for patients using currently available commercial vitamin D kits might be significantly overestimated. This may have practical implications for current supplementation strategies and guidelines.

## 5. Conclusions

Our main finding is that the CC SNP3 gene had statistically significant influence on vitamin D3 levels. This finding, however, needs more research because the reasons for it are not clear at present. Vitamin D and/or calcium supplements, however, had no effect on vitamin D epimer and metabolite concentration before or after adjusting for the presence of the four VDR genotypes and alleles. Although clinical evidence at present does not exist for the routine quantification of vitamin D epimers and metabolites, there is a need to be aware that the presence of vitamin D epimers and metabolite might lead to an overestimation of vitamin D values. Future research is also needed to study the effects of a higher dose of vitamin D supplementation to reach higher levels, particularly in high-risk populations, to see if this will affect vitamin D epimers and metabolites.

## Figures and Tables

**Table 1:** Baseline characteristics at trial entry by randomization group, mean (SD) unless stated otherwise.

		Vitamin D3 (*n* = 68)	Vitamin D3 + Calcium (*n* = 68)	Calcium (*n* = 75)	Placebo (*n* = 66)	*p* Value
Age (years)		42 (11)	41 (13)	41 (13)	41 (13)	0.89
Gender, *n* (%)	Female	55 (78)	55 (73)	48 (73)	48 (70)	0.28
Smoking, *n* (%)						0.94
	Yes	8 (11)	10 (13)	7 (11)	10 (15)	
	Occasionally	5 (7)	3 (4)	2 (3)	2 (3)	
	No	55 (79)	63 (82)	55 (83)	55 (80)	
Diabetes mellitus, *n* (%)		7 (10)	10 (13)	12 (18)	17 (25)	0.02
Hypertension, *n* (%)		8 (11)	11 (15)	9 (14)	14 (20)	0.14
BMI		29.3 (5)	28.7 (5.5)	29 (6)	28.6 (5)	0.99
Physical activity, *n* (%)						0.65
	Very active	18 (25)	17 (23)	12 (18)	8 (12)	
	Moderate	41 (59)	47 (62)	37 (57)	49 (72)	
	Not active	11 (16)	17 (22)	16 (25)	11 (16)	
Systolic blood pressure, (mm/Hg)		118 (8)	120 (9)	116 (10)	122 (14)	0.35
Diastolic blood pressure, (mm/Hg)		75 (6)	79 (5)	76 (6)	77 (6)	0.28
Cholesterol, (mmol/L)		7 (10)	8 (11)	4 (6)	14 (20)	0.08
Glucose, (mmol/L)		6.5 (2)	6.2 (2.5)	6.2 (2.3)	6.3 (2.5)	0.88
HbA1c		5.9 (1)	5.8 (0.9)	5.8 (1)	5.8 (0.9)	0.97
Urea, (mmol/L)		4.4 (1.7)	3.9 (1.2)	3.9 (1.2)	4.2 (1.6)	0.07
Creatinine, (mmol/L)		52 (12)	52 (12)	51 (12)	53 (15)	0.69
Vitamin D, (ng/mL)		23 (9)	19 (11)	25 (11)	25 (11)	0.58

All values fall within normal limits.

**Table 2:** Vitamin D epimer and metabolite concentrations according to presence or absence of genotype and allele distribution of RS1544410_SNP1_Bsml VDR.

	AA		GG		AG		A		G	
	Yes	No	Yes	No	Yes	No	Yes	No	Yes	No
Vitamin D3 (ng/mL)	12.49 (5.61)	12.52 (6.36)	11.85 (5.29)	12.92 (6.67)	13.15 (7.18)	12.07 (5.38)	12.93 (6.71)	11.83 (5.24)	12.53 (6.37)	12.46 (5.56)
Vitamin D2 (ng/mL)	5.72 (1.15)	5.87 (1.17)	5.84 (1.18)	5.84 (1.16)	5.90 (1.17)	5.80 (1.16)	5.84 (1.17)	5.84 (1.17)	5.87 (1.17)	5.72 (1.14)
25OHD3 (ng/mL)	3.80 (0.82)	3.98 (1.43)	3.83 (0.89)	4.02 (1.52)	4.13 (1.77)	3.82 (0.86)	4.02 (1.53)	3.83 (0.88)	3.98 (1.43)	3.80 (0.81)
3-epi-25OHD3 (ng/mL)	1.29 (0.21)	1.31 (0.25)	1.29 (0.25)	1.31 (0.23)	1.32 (0.25)	1.29 (0.24)	1.31 (0.24)	1.29 (0.25)	1.31 (0.25)	1.29 (0.21)
7αC4 * (ng/mL)	11.99 (11.74)	(11.93) 11.43	12.13 (11.86)	11.83 (11.27)	11.80 (11.11)	12.04 (11.76)	11.89 (11.32)	12.02 (11.77)	11.95 (11.44)	11.89 (11.67)

*p* < 0.05 for difference between those with and those without the genotype and allele 4 VDR gene polymorphism. * 7αC4 = 7-α-Hydroxy-4-cholesten-3-one.

**Table 3:** Vitamin D epimer and metabolite concentrations according to presence or absence of genotype and allele distribution of RS731236_SNP2_Taql gene polymorphism.

	AA		GG		AG		A		G	
	Yes	No	Yes	No	Yes	No	Yes	No	Yes	No
Vitamin D3 (ng/mL)	12.06 (6.09)	12.72 (6.26)	11.61 (4.54)	12.73 (6.52)	13.17 (6.79)	11.88 (5.52)	12.74 (6.54)	11.59 (4.50)	12.74 (6.27)	12.04 (6.06)
Vitamin D2 (ng/mL)	5.81 (1.21)	5.85 (1.15)	5.69 (1.20)	5.88 (1.16)	5.92 (1.13)	5.77 (1.19)	5.88 (1.16)	5.70 (1.19)	5.85 (1.15)	1.20 (5.81)
25OHD3 (ng/mL)	3.84 (1.08)	3.99 (1.42)	3.88 (1.03)	3.96 (1.39)	4.03 (1.55)	3.86 (1.05)	3.96 (1.39)	3.89 (1.02)	3.99 (1.42)	3.84 (1.07)
3-epi-25OHD3 (ng/mL)	1.33 (0.27)	1.29 (0.23)	1.29 (0.22)	1.30 (0.25)	1.29 (0.23)	1.31 (0.25)	1.31 (0.25)	1.29 (0.22)	1.29 (0.23)	1.33 (0.27)
7αC4 (ng/mL)	10.68 (10.24)	12.51 (11.97)	12.12 (12.89)	11.90 (11.14)	12.71 (11.67)	11.19 (11.27)	11.92 (11.16)	12.00 (12.80)	12.54 (11.99)	10.62 (10.19)

*p* < 0.05 for difference between those with and those without the genotype and allele 4 VDR gene polymorphisms.

**Table 4:** Vitamin D epimer and metabolite concentrations according to presence or absence of genotype and allele distribution of RS7975232_SNP3_Apal gene polymorphism.

	AA		CC		AC		A		C	
	Yes	No	Yes	No	Yes	No	Yes	No	Yes	No
Vitamin D3 (ng/mL)	13.16 (6.76)	12.01 (5.70)	11.28 (3.43)	12.77 * (6.60)	12.34 (6.45)	12.63 (6.06)	12.78 (6.61)	11.26 (3.40)	12.02 (5.72)	13.14 (6.74)
Vitamin D2 (ng/mL)	5.77 (1.03)	5.90 (1.27)	5.64 (1.05)	5.88 (1.19)	6.01 (1.34)	5.73 (1.03)	5.88 (1.19)	5.64 (1.04)	5.90 (1.27)	5.77 (1.02)
25OHD3 (ng/mL)	4.00 (1.51)	3.91 (1.15)	3.74 (0.74)	3.987 (1.41)	3.98 (1.29)	3.93 (1.35)	3.99 (1.41)	3.74 (0.73)	3.90 (1.16)	4.00 (1.50)
3-epi-25OHD3 (ng/mL)	1.33 (0.23)	1.28 (0.25)	1.32 (0.26)	1.30 (0.24)	1.27 (0.25)	1.32 (0.23)	1.30 (0.24)	1.32 (0.25)	1.28 (0.25)	1.32 (0.22)
7αC4 (ng/mL)	12.18 (11.34)	11.75 (11.60)	13.18 (13.29)	11.69 (11.09)	11.19 (10.86)	12.41 11.85	11.72 (11.11)	13.18 (13.03)	11.79 (11.63)	12.13 (11.31)

* *p* < 0.05 for difference between those with and those without the genotype and allele 4 VDR gene polymorphisms.

**Table 5:** Vitamin D epimer and metabolite concentrations according to presence or absence of genotype and allele distribution of RS2228570_SNP4_Fok1 gene polymorphism.

	AA		GG		AG		A		G	
	Yes	No	Yes	No	Yes	No	Yes	No	Yes	No
Vitamin D3 (ng/mL)	11.21 (3.89)	12.68 (6.43)	12.38 (6.85)	12.62 (5.70)	12.99 (6.04)	12.12 (6.33)	12.13 (6.35)	12.97 (6.02)	12.64 (6.41)	11.59 (4.42)
Vitamin D2 (ng/mL)	5.57 (1.11)	5.88 (1.17)	5.75 (1.10)	5.91 (1.21)	5.99 (1.23)	5.72 (1.10)	5.71 (1.10)	5.99 (1.23)	5.88 (1.17)	5.59 (1.08)
25OHD3 (ng/mL)	3.95 (1.53)	3.95 (1.30)	3.84 (0.96)	4.03 (1.54)	4.05 (1.55)	3.86 (1.10)	3.86 (1.10)	4.05 (1.55)	3.95 (1.30)	3.95 (1.48)
3-epi-25OHD3 (ng/mL)	1.24 (0.20)	1.31 (0.25)	1.33 (0.25)	1.28 (0.23)	1.30 (0.24)	1.31 (0.24)	1.31 (0.24)	1.30 (0.24)	1.31 (0.25)	1.24 (0.19)
7αC4 (ng/mL)	12.80 (9.44)	11.83 (11.72)	12.37 (12.96)	11.62 (10.26)	11.36 (10.50)	12.42 (12.23)	12.47 (12.26)	11.32 (10.47)	11.87 (11.76)	12.46 (9.26)

*p* < 0.05 for difference between those with and those without the genotype and allele 4 VDR gene polymorphisms.

**Table 6:** Vitamin D epimer and metabolite outcomes by randomized group, mean (SD) unless stated otherwise.

Clinical Variable	Vitamin D3 (*n* = 68)		RG2 Vitamin D3 + Calcium (*n* = 68)		RG3 Calcium (*n* = 75)		RG4 Placebo (*n* = 66)	
	Baseline	Follow Up	Baseline	Follow Up	Baseline	Follow Up	Baseline	Follow Up
Vitamin D3	12.37 (5.82)	37.41 (21.21)	11.81 (5.84)	37.08 (24.02)	13.43 (6.67)	41.467 (29.14)	12.35(6.41)	46.15 (36.22)
Vitamin D2	5.71 (1.10)	6.97 (1.71)	5.66 (1.05)	6.78 (1.36)	6.05 (1.12)	7.17 (1.57)	5.93 (1.36)	7.77 (2.71)
25 OHD3	3.94 (1.18)	7.34 (3.44)	3.84 (1.28)	7.83 (4.36)	4.20 (1.78)	6.82 (3.60)	3.78 (0.79)	7.62 (3.89)
25OHD2	4.18 (0.85)	6.62 (2.90)	3.58 (0.46)	3.14 (0.24)	4.26 (0.84)	8.89 (6.28)	6.19 (4.04)	6.12 (3.12)
1α,25(OH)2D3	5.63 (2.25)	6.19 (3.12)	5.96 (3.12)	5.81 (2.79)	4.85 (2.61)	6.18 (2.90)	5.06 (2.32)	6.79 (3.79)
3-epi-25OHD3	1.28 (0.20)	2.21 (0.92)	1.24 (0.23)	1.97 (1.06)	1.42 (0.24)	1.73 (0.93)	1.35 (0.21)	2.19 (1.29)
7αC4	12.17 (9.49)	11.90 (8.27)	10.97 (10.92)	14.63 (13.36)	11.50 (11.52)	11.52 (8.57)	13.21 (13.75)	14.71 (10.46)

*p* value < 0.05 for between-group difference in cumulative changes at 6 months adjusting for the presence of the 4 VDR genotype and allele gene polymorphisms.

## Data Availability

The original contributions presented in the study are included in the article, further inquiries can be directed to the corresponding author.
